# Supplementary material for: Integrins mediate symbiont-specific uptake in cnidarian larvae
Source: EMBO Rep. 2025 Dec 16;27(2):291–310. doi: 10.1038/s44319-025-00645-9 (PMC12852126; doi:10.1038/s44319-025-00645-9)
Supplement: Supplementary file 1 — Table EV1 [file 44319_2025_645_MOESM1_ESM.docx]

Table EV1. Plasmids used in (F)ISH and cell-culture experiments

| Internal number | Insert | Backbone | Fluorophore | Addgene number |
| --- | --- | --- | --- | --- |
| P-0062 |  | pCRII-TOPO/8c + ASCI&PacI restriction sites |  |  |
| P-0251 | Aiptasia Probe ITA1 | pCRII-TOPO/8c + ASCI&PacI restriction sites |  |  |
| P-0266 | Human Alpha-V-Integrin | mEmerald-N1 | mEmerald | 53985 |
| P-0268 | Mouse Integrin-Beta3 | mEmerald-N1 | mEmerald | 54130 |
| P-0273 | farnesylated eGFP | pEGFP (clonetech) obtained from Ary Shalizi, Stanford University | eGFP |  |
| P-0299 | Human Alpha-V-Integrin |  | C-term-split-YFP |  |
| P-0300 | Integrin-Beta3 |  | N-term-split-YFP |  |
| P-0301 | Empty | pCS2+ gift from Sergio Acebron, Heidelberg University |  |  |
| P-0302 | Integrin-Beta3 (D119A) |  | N-term-split-YFP |  |
| P-0303 | Integrin-Beta3 (D119A+D217A) |  | N-term-split-YFP |  |
