# Supplementary material for: Integrins mediate symbiont-specific uptake in cnidarian larvae
Source: EMBO Rep. 2025 Dec 16;27(2):291–310. doi: 10.1038/s44319-025-00645-9 (PMC12852126; doi:10.1038/s44319-025-00645-9)
Supplement: Supplementary file 2 — Table EV2 [file 44319_2025_645_MOESM2_ESM.docx]

Table EV2. Primers used for construction of plasmids

| Internal number | Name | Sequence | Restriction Enzyme | Final plasmid | Template | Cloning methode |
| --- | --- | --- | --- | --- | --- | --- |
| O-0849 | IntA1-ISH-probe_fw | ATAGGCGCGCCCAGATAACAACAATGCTTTGGTATC | AscI | P-0251 | CC7 cDNA | Restriction cloning |
| O-0850 | IntA1-ISH-probe_rv | GACTTAATTAAGTCCAGTTATAACAACTGTCTAGTAC | PacI | P-0251 | CC7 cDNA | Restriction cloning |
| O-1392 | Split_YFP_C-term_rv | CGGCCGCTTACTTGTACAGCTCGTC |  | P-299 |  | NEBuilder® HiFi DNA Assembly |
| O-1395 | Split_YFP_C-term_fw | GGTCGCCACCTACCCATACGATGTTCC |  | P-299 |  | NEBuilder® HiFi DNA Assembly |
| O-1397 | Split_YFP_N-term_fw | GGTCGCCACCATGGTGAGCAAGGGC |  | P-300 |  | NEBuilder® HiFi DNA Assembly |
| O-1399 | Split_YFP_N-term_rv | CGGCCGCTTAAAGATCCTCCTCAGAAATC |  | P-300 |  | NEBuilder® HiFi DNA Assembly |
| O-1408 | AlphaV+backbone_fw | GCTGTACAAGTAAGCGGCCGCGACTC |  | P-299 | P-0266 | NEBuilder® HiFi DNA Assembly |
| O-1409 | AlphaV+backbone_rv | CGTATGGGTAGGTGGCGACCGGTGG |  | P-299 | P-0266 | NEBuilder® HiFi DNA Assembly |
| O-1422 | Beta3+backbone_fw | GGAGGATCTTTAAGCGGCCGCGACTC |  | P-300 | P-0268 | NEBuilder® HiFi DNA Assembly |
| O-1423 | Beta3+backbone_rv | TGCTCACCATGGTGGCGACCGGTCC |  | P-300 | P-0268 | NEBuilder® HiFi DNA Assembly |
| O-1662 | Human ITB3 D119A mutation fw | CTACTTGATGgccCTGTCTTTCTC |  | P-0302 | P-0300 | Site-directed Mutagenisis |
| O-1663 | Human ITB3 D119A mutation rv | TAGATGTCCACGGGGTAA |  | P-0302 | P-0300 | Site-directed Mutagenisis |
| O-1664 | Human ITB3 D217A mutation fw | CCGTAATCGAgctGCCCCAGAGG |  | P-0303 | P-0302 | Site-directed Mutagenisis |
| O-1665 | Human ITB3 D217A mutation rv | GACACGCTCTGTTTCTTCACTTC |  | P-0303 | P-0302 | Site-directed Mutagenisis |
