# Supplementary figures and images for: Integrins mediate symbiont-specific uptake in cnidarian larvae
Source: EMBO Rep. 2025 Dec 16;27(2):291–310. doi: 10.1038/s44319-025-00645-9 (PMC12852126; doi:10.1038/s44319-025-00645-9)

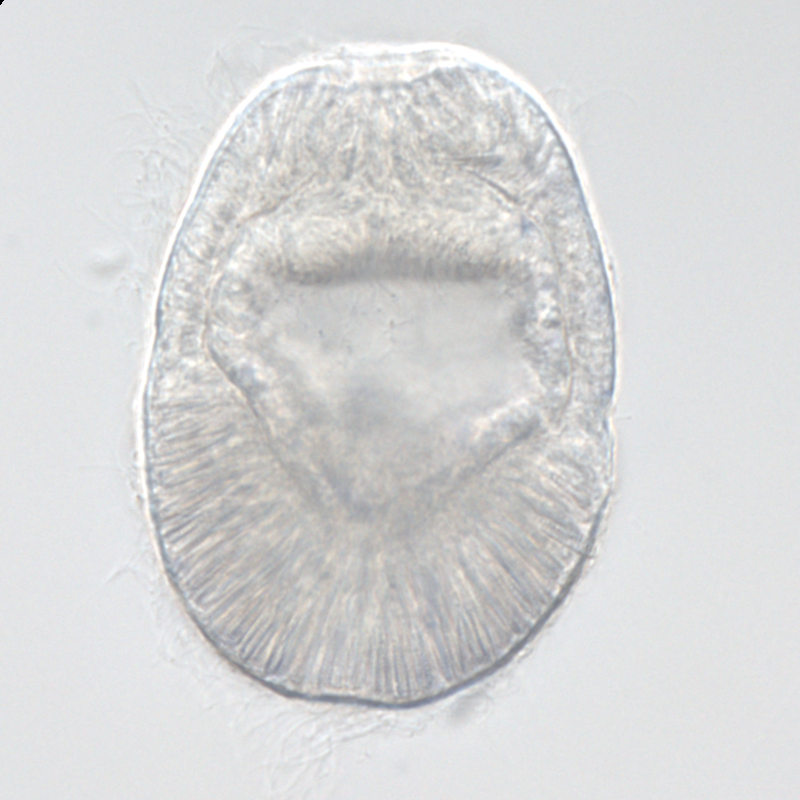

Supplement: Supplementary file 6 — Source data Fig. 2 [file 44319_2025_645_MOESM6_ESM.zip › EMBOR-2025-61328V2_SourceDataFig2/Fig2B/cropped_2018-10-28_Alpha4 sense 40x 10.tif]

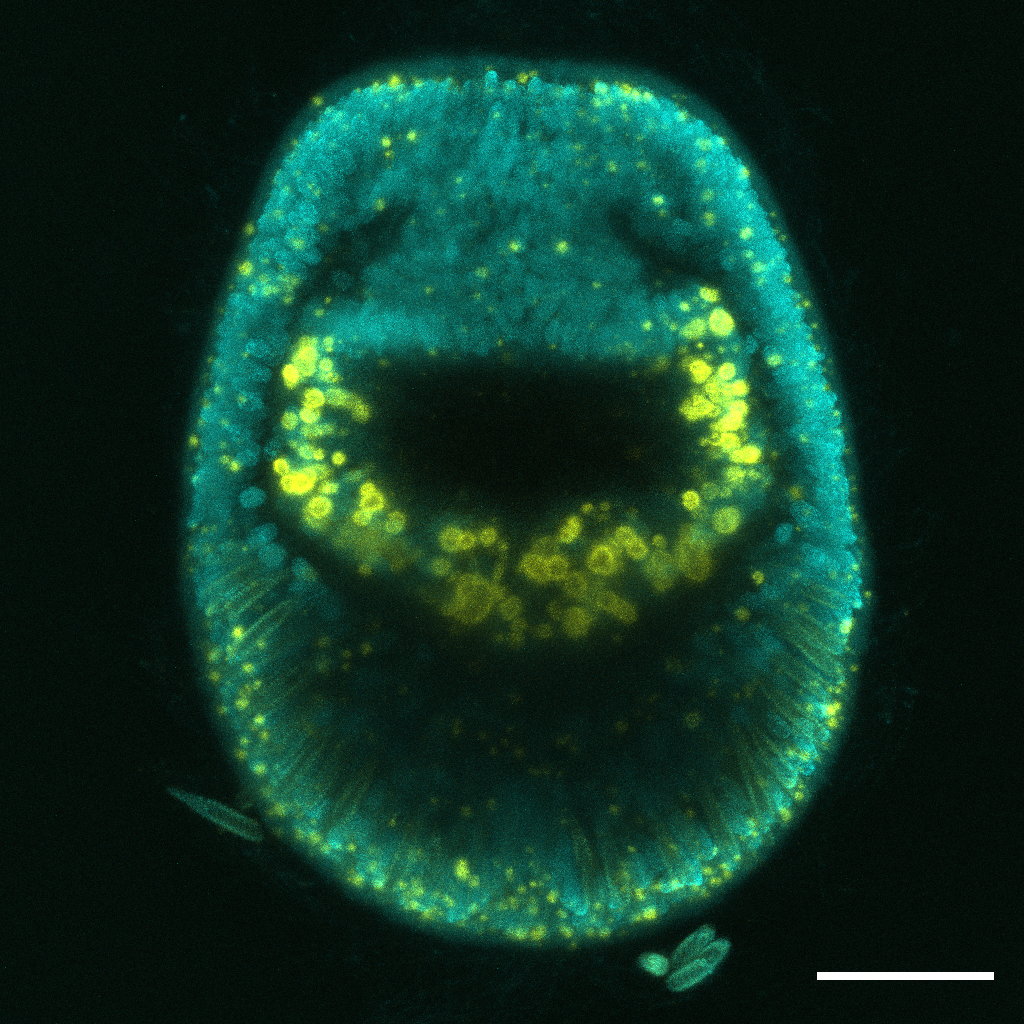

Supplement: Supplementary file 6 — Source data Fig. 2 [file 44319_2025_645_MOESM6_ESM.zip › EMBOR-2025-61328V2_SourceDataFig2/Fig2E/MAX_20181027 FISH intalpha 1 4.lif - IntAlpha4 AS 2 whole.tif (CY)_20┬╡m.tif]

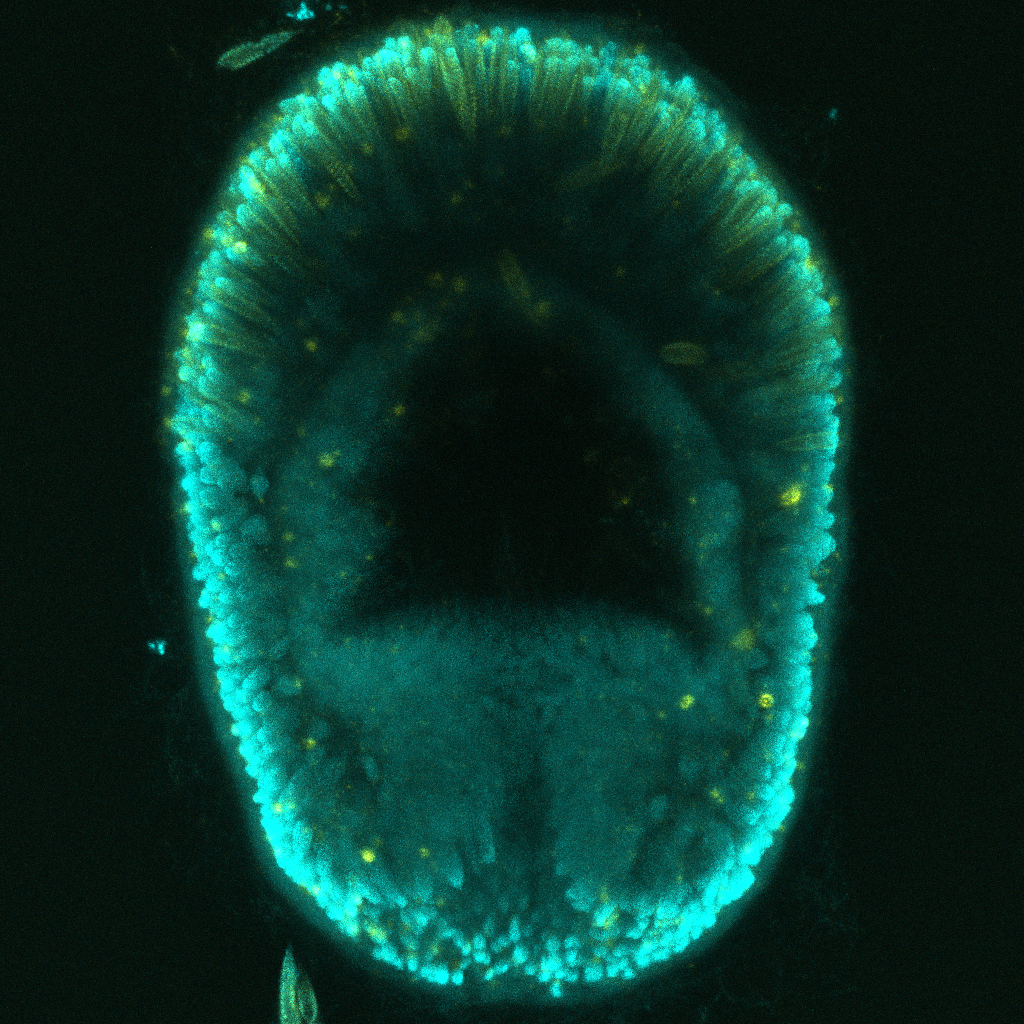

Supplement: Supplementary file 6 — Source data Fig. 2 [file 44319_2025_645_MOESM6_ESM.zip › EMBOR-2025-61328V2_SourceDataFig2/Fig2D/MAX_20181027 FISH intalpha 1 4.lif - IntAlpha4 sense 5 whole.tif (CY).tif]

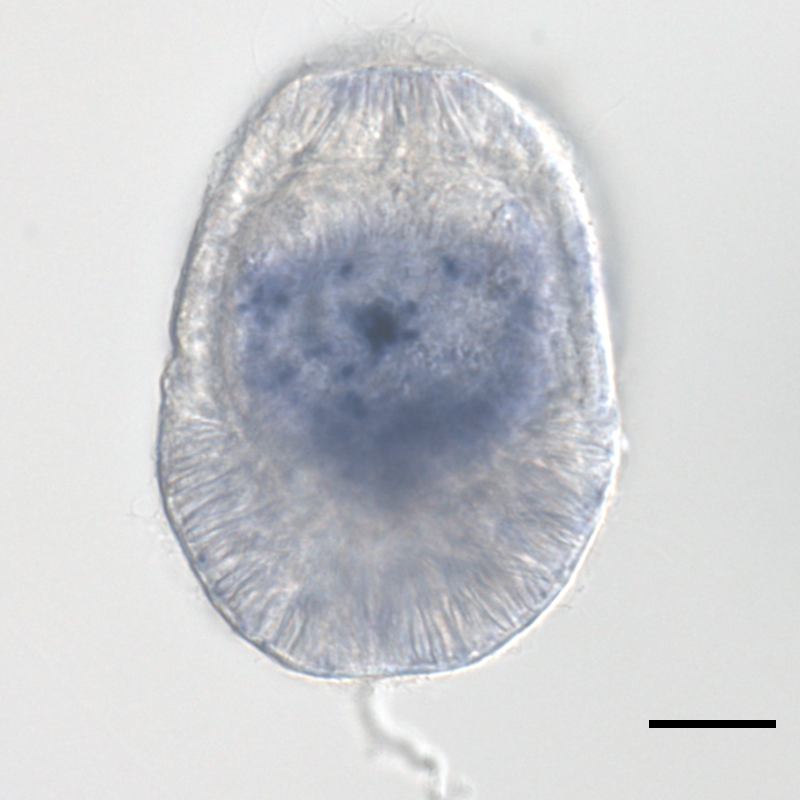

Supplement: Supplementary file 6 — Source data Fig. 2 [file 44319_2025_645_MOESM6_ESM.zip › EMBOR-2025-61328V2_SourceDataFig2/Fig2C/cropped_2018-10-28_Alpha4 AS 40x 3-1_20micron_scalebar.tif]

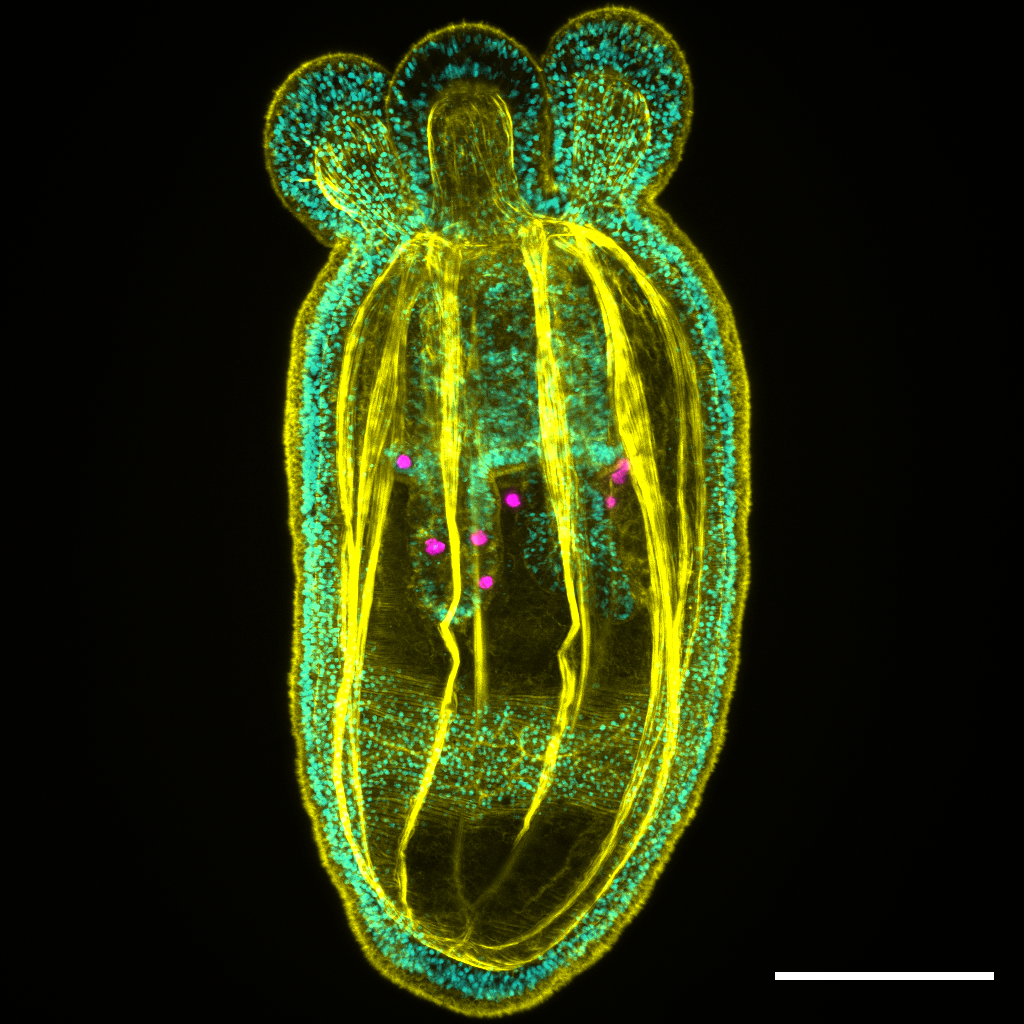

Supplement: Supplementary file 7 — Source data Fig. 3 [file 44319_2025_645_MOESM7_ESM.zip › EMBOR-2025-61328V2_SourceDataFig3/Fig3I/MAX_20210126_RGDBlocking_RGD_SSB01.lif - RGD0_SSB01_001.tif (RGB)_100┬╡m.tif]

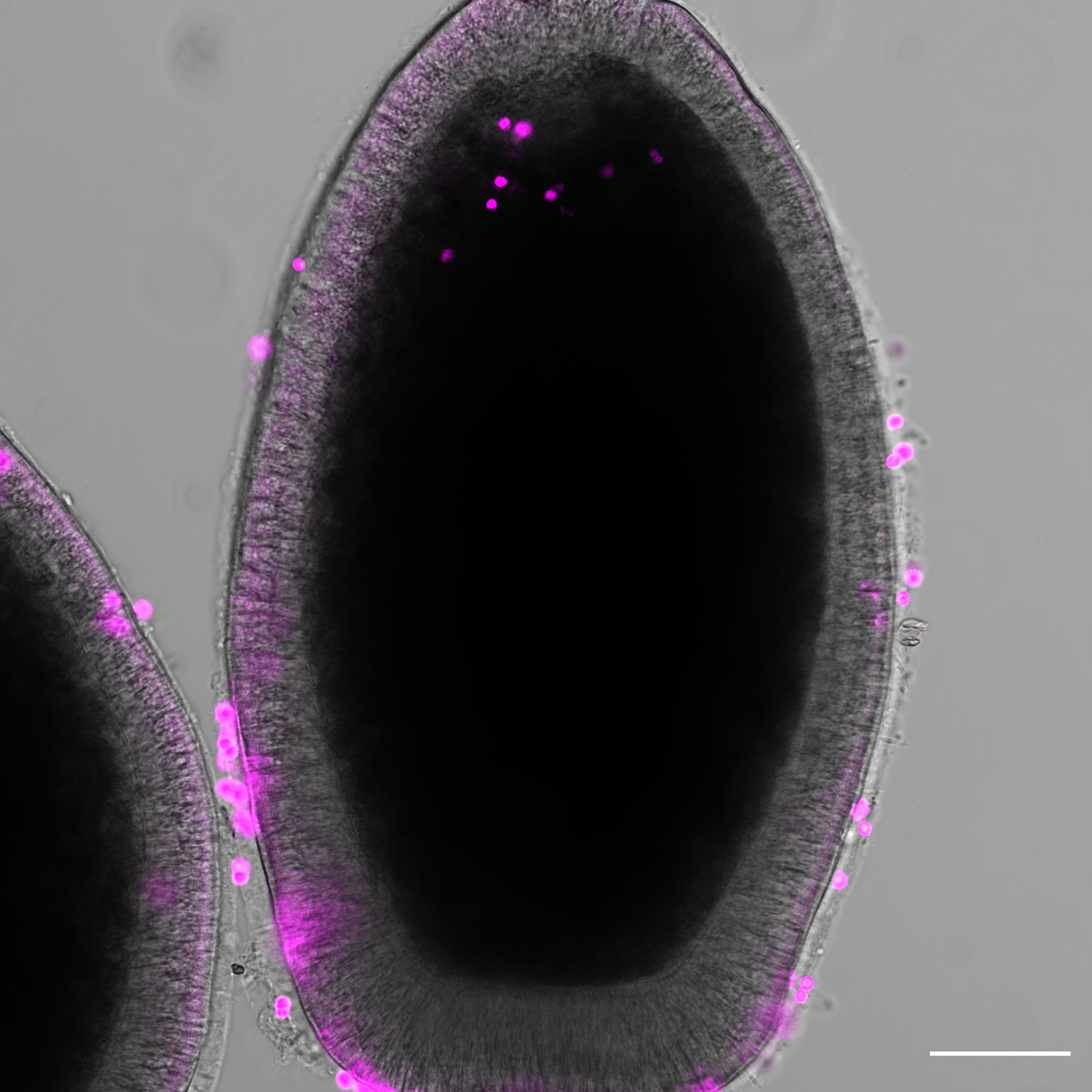

Supplement: Supplementary file 7 — Source data Fig. 3 [file 44319_2025_645_MOESM7_ESM.zip › EMBOR-2025-61328V2_SourceDataFig3/Fig3G/20190703 acropora bead larvae.lif - ad85 21 15x.tif (M)_100┬╡m.tif]

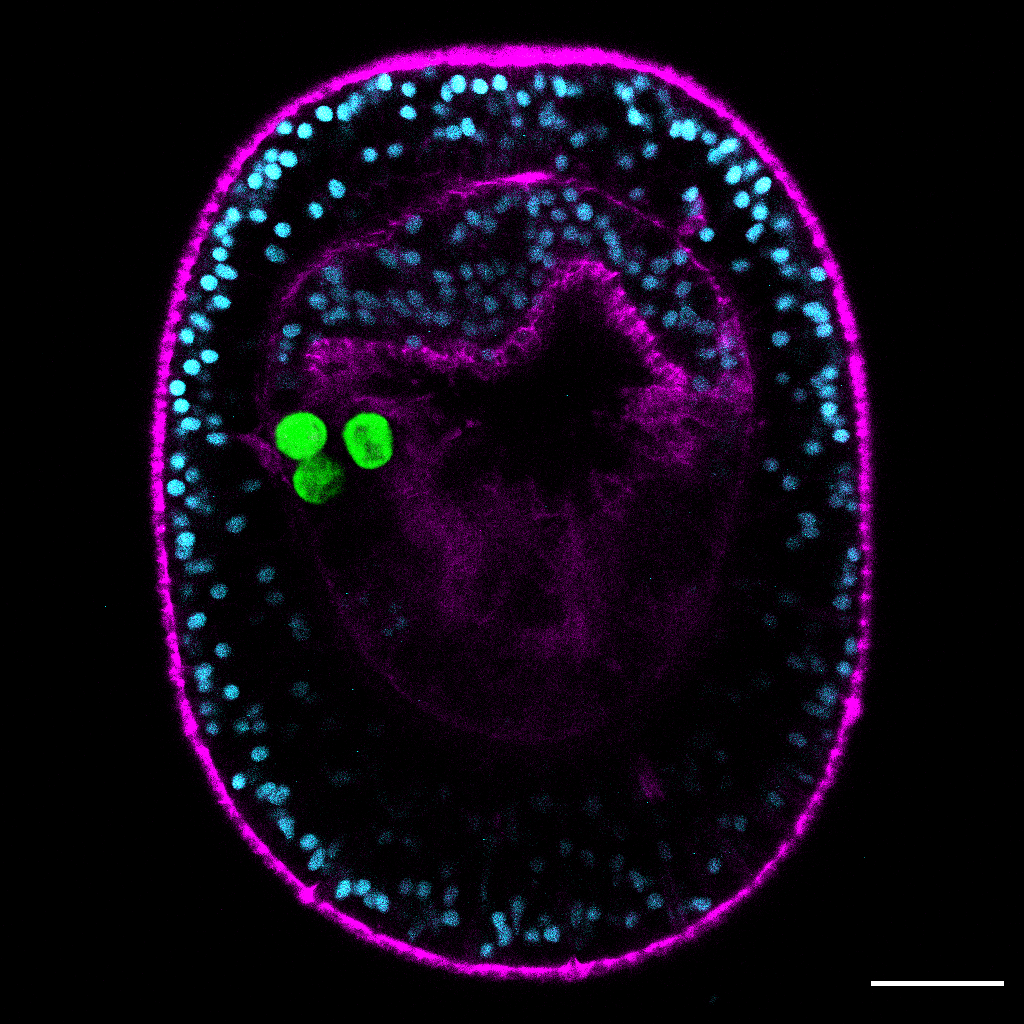

Supplement: Supplementary file 7 — Source data Fig. 3 [file 44319_2025_645_MOESM7_ESM.zip › EMBOR-2025-61328V2_SourceDataFig3/Fig3A/20190508 RGD DGR block B01 Noc Cve.lif - BO1 DGR 2 whole.tif (CMG)_20┬╡m.tif]

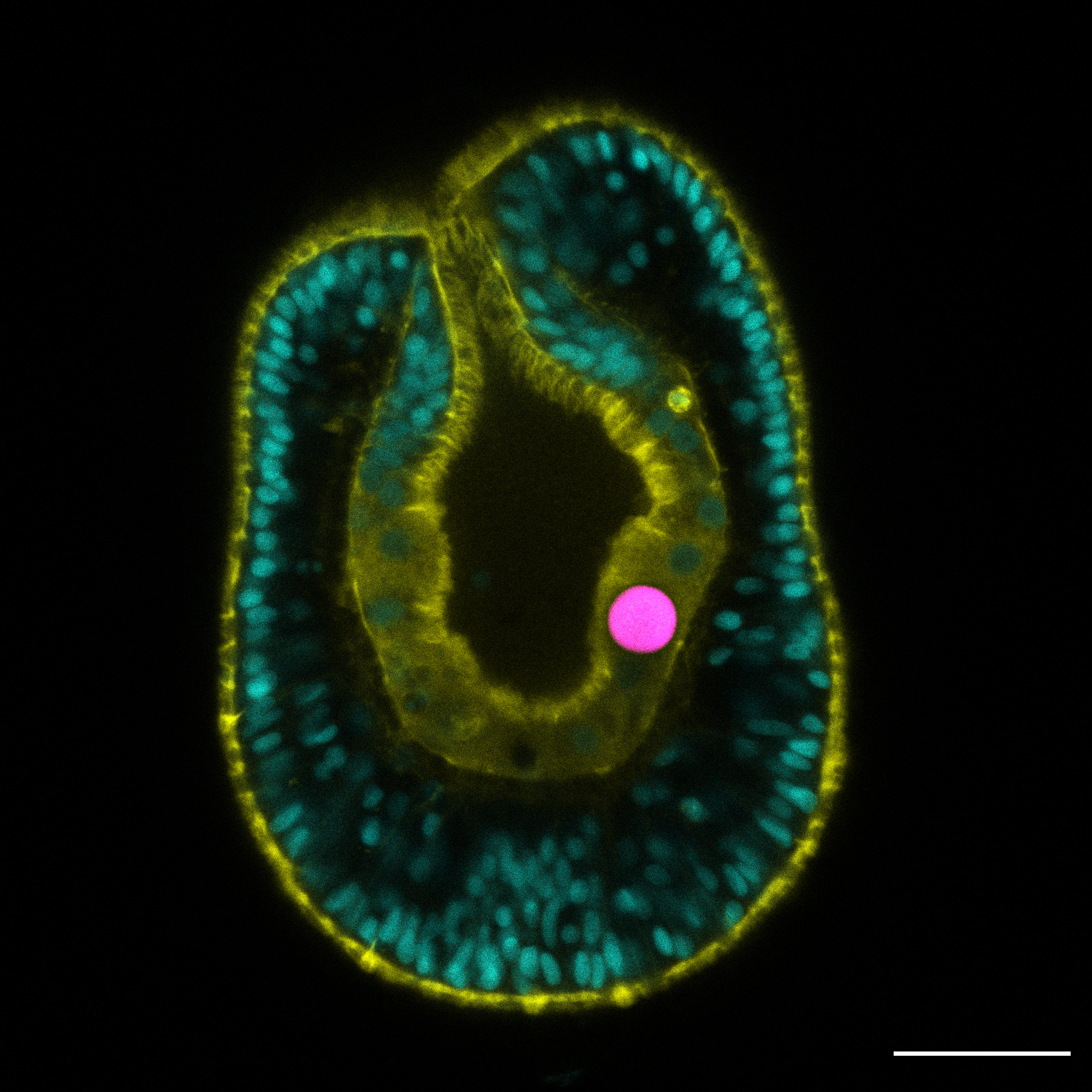

Supplement: Supplementary file 7 — Source data Fig. 3 [file 44319_2025_645_MOESM7_ESM.zip › EMBOR-2025-61328V2_SourceDataFig3/Fig3C/20190512 RGD beads.lif - RGD bead 3 1x5.tif (CYM)_20┬╡m.tif]

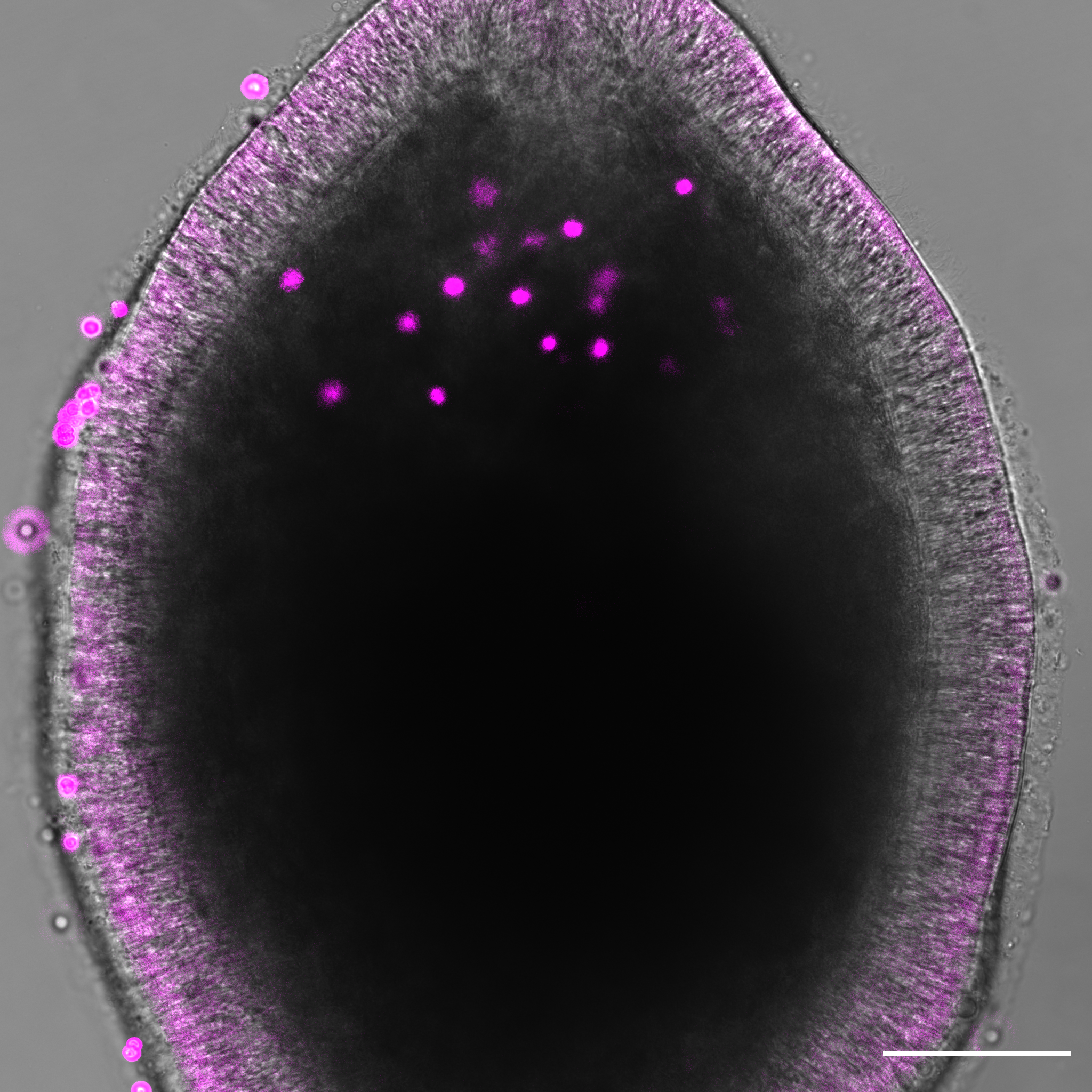

Supplement: Supplementary file 7 — Source data Fig. 3 [file 44319_2025_645_MOESM7_ESM.zip › EMBOR-2025-61328V2_SourceDataFig3/Fig3E/20190725 acropora peptide block.lif - 57 20 1x.tif (M)_100┬╡m.tif]

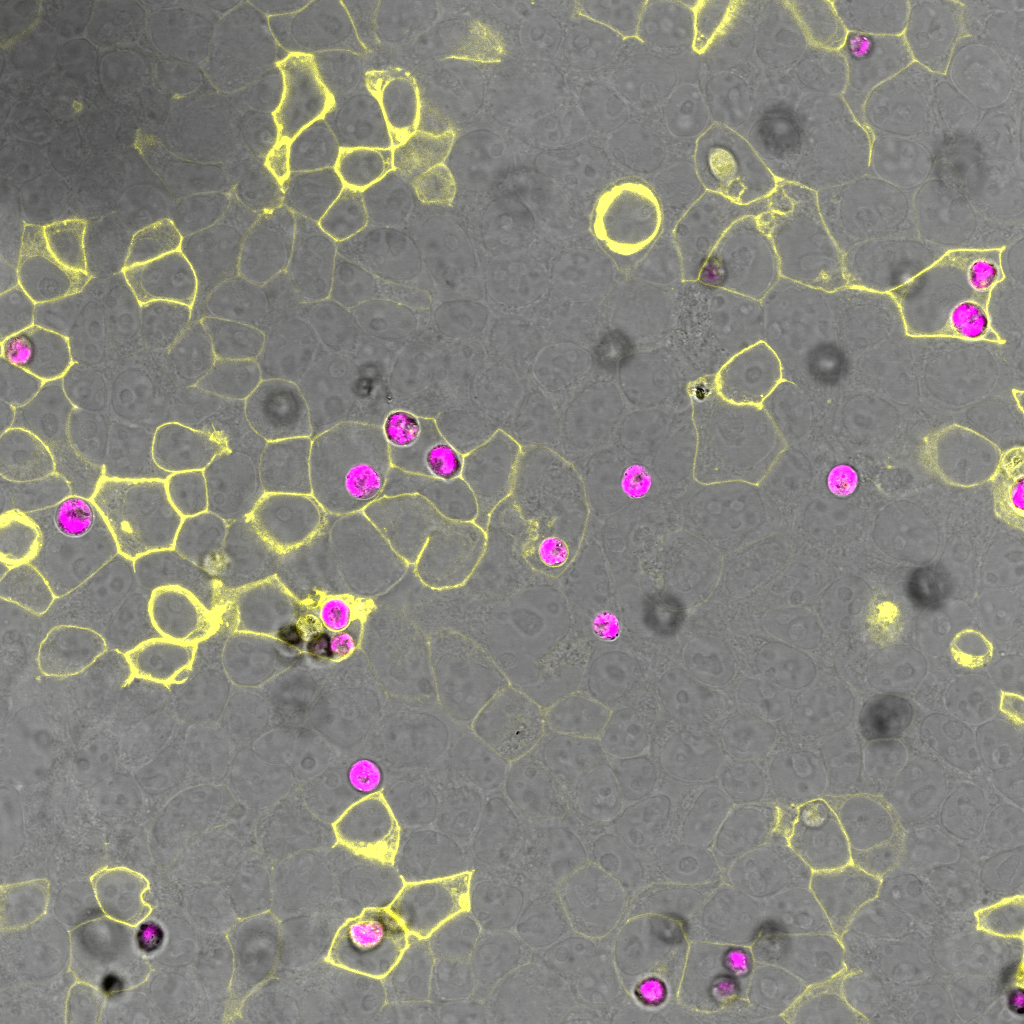

Supplement: Supplementary file 8 — Source data Fig. 4 [file 44319_2025_645_MOESM8_ESM.zip › EMBOR-2025-61328V2_SourceDataFig4/Fig4A/20210219_HekUptake_aVCb3N_YFP_SSB01_90minInf.lif - aVCb3N_90minInf_Inf48hpt_SSB01Conc.2_002-1.tif (RGB).tif]

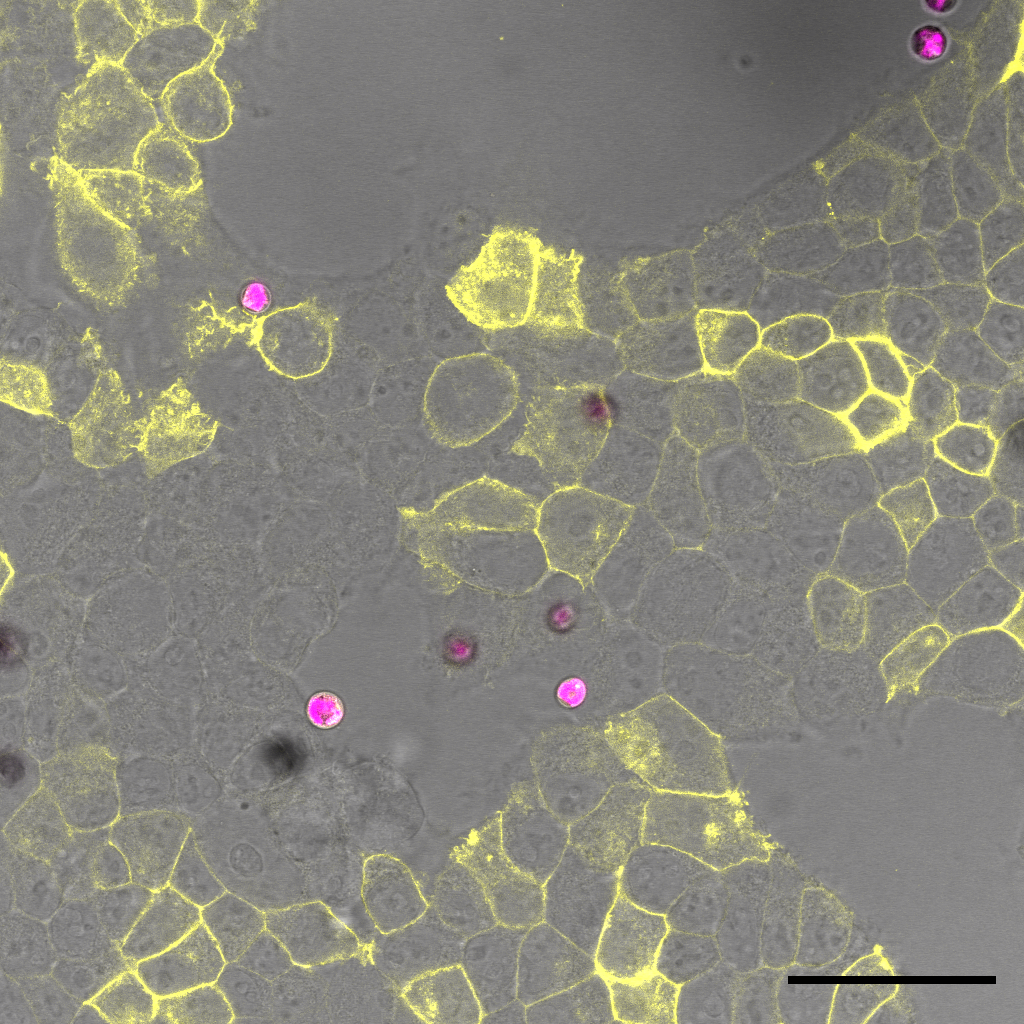

Supplement: Supplementary file 8 — Source data Fig. 4 [file 44319_2025_645_MOESM8_ESM.zip › EMBOR-2025-61328V2_SourceDataFig4/Fig4C/20210521_HekUptake_fGFP_SSB01_90min.lif - 20210521_1_fGFP_90min_Inf48hpt_SSB01Conc.3_002-1.tif (RGB).tif]

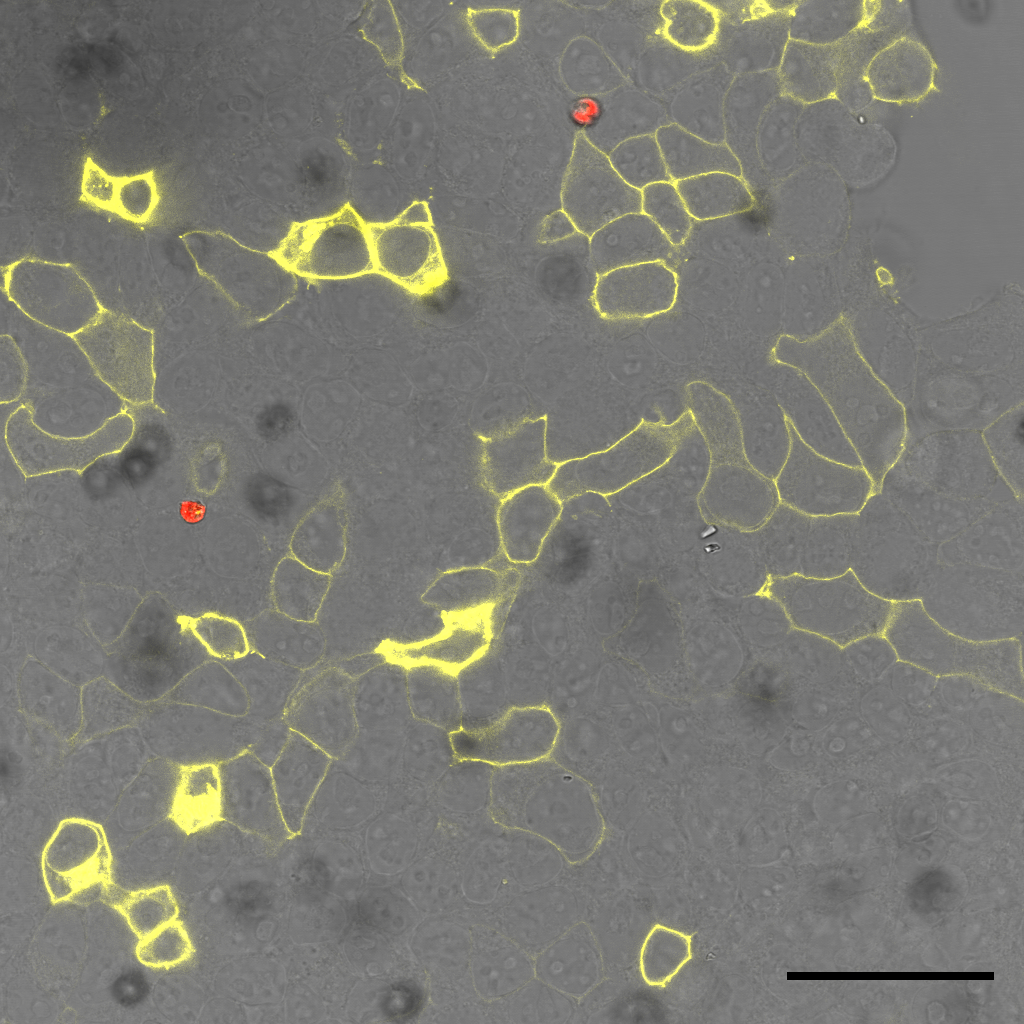

Supplement: Supplementary file 8 — Source data Fig. 4 [file 44319_2025_645_MOESM8_ESM.zip › EMBOR-2025-61328V2_SourceDataFig4/Fig4B/20210430_HekUptake_aVCD217A_SSB01_90min.lif - 1_aVCD217A_90min_Inf48hpt_SSB01Conc.3_003-1_red_50┬╡m.tif]
