# Supplementary material for: Integrins mediate symbiont-specific uptake in cnidarian larvae
Source: EMBO Rep. 2025 Dec 16;27(2):291–310. doi: 10.1038/s44319-025-00645-9 (PMC12852126; doi:10.1038/s44319-025-00645-9)
Supplement: Supplementary file 9 — Expanded View Figures [file 44319_2025_645_MOESM9_ESM.pdf]

Expanded View Figures

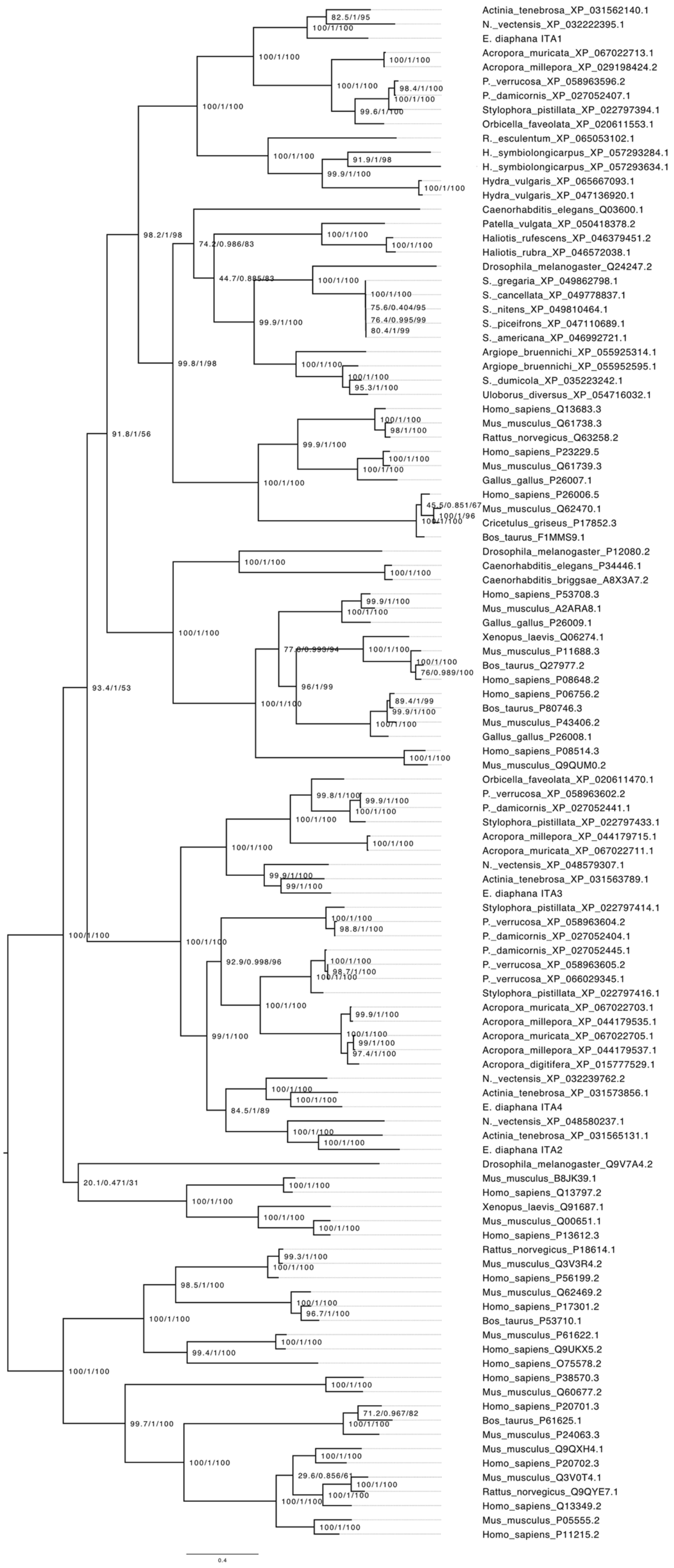

Figure EV1. Phylogenetic tree of integrin α proteins prior to collapse of subtrees and rotation of nodes.

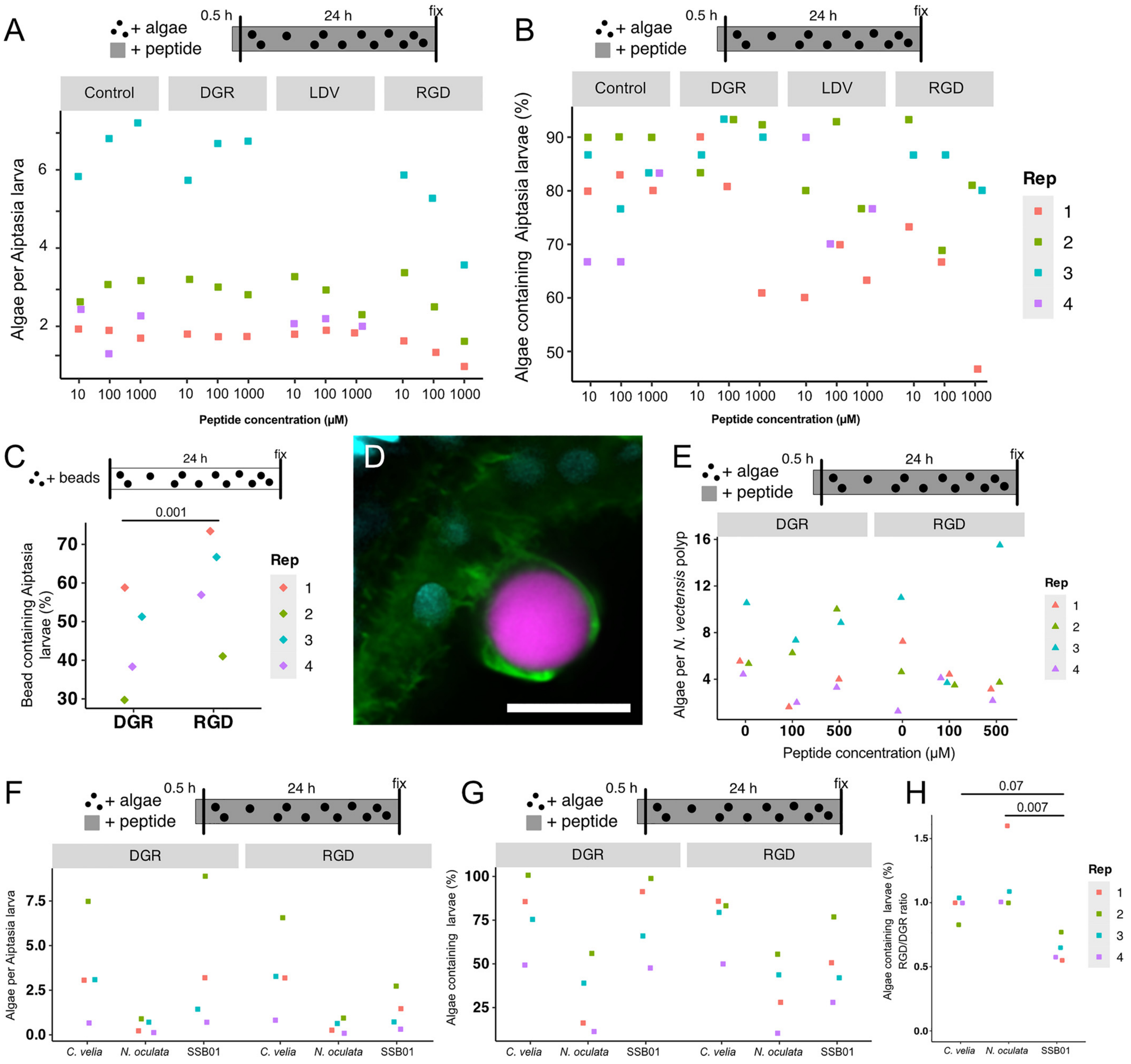

Figure EV2. Raw and extended data for Fig. 3 RGD-integrins facilitate symbiont uptake in symbiotic cnidaria.

(**A**, **B**) Aiptasia larvae were exposed to control peptide DGR, integrin ligand RGD or integrin ligand LDV for 30 min then exposed to symbionts (*B. minutum*, SSB01) for 24 h. $n = 3$. (**A**) SSB01 internalized by Aiptasia larvae. (**B**) Percent of Aiptasia larvae that phagocytosed SSB01. (**C**) Percent of Aiptasia larvae that phagocytosed peptide-coated beads. Beads were coated with either control peptide DGR or integrin ligand RGD then incubated with Aiptasia larvae for 24 h. $n = 4$. (**D**) Aiptasia larvae exposed to inert beads coated with RGD peptides and imaged after 48 h of exposure. Green = actin, Cyan = DNA, pink = bead. Scale bar = 10 μm. (**E**) SSB01 per *N. vectensis* polyp after a 24 h incubation and exposure to either the control peptide DGR or integrin ligand RGD. $n = 4$. (**F**) Algae per Aiptasia larvae and (**G**) Percent of Aiptasia larvae with algae inside upon RGD or DGR treatment (1000 μM peptide). $n = 4$. (**H**) Percent of larvae from G normalized by taking the ratio between RGD to DGR treatment. A ratio of less than 1 means RGD treatment has lower algae uptake than DGR. $n = 4$. For all plots, biological replicates are colored to highlight trends and natural variation between replicates. For (**A**, **B**, **E–H**) ANOVA was used to determine significance. Only significant differences are shown. For (**C**), significance was found via paired *t* test.

 © The Author(s)

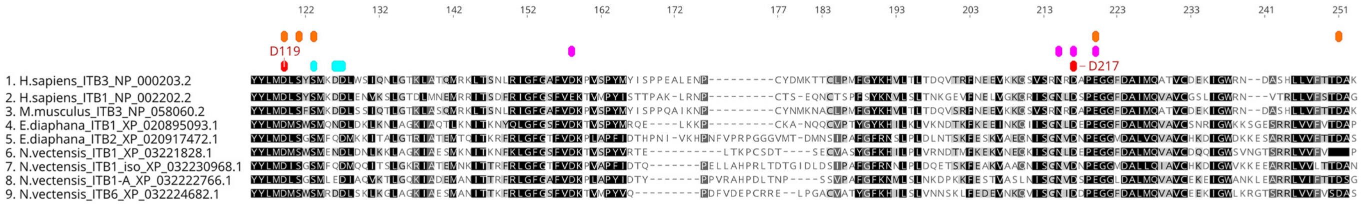

Figure EV3. Active sites of RGD-binding integrin and mutations used for this study.

Highly conserved residues are highlighted in black and as conservation decreases this goes from gray to white. Conserved active sites are noted above with the colored labels. Orange = MIDAS. Turquoise = ADMIDAS. Pink = LIMBS. Red = used mutations to disrupt RGD binding.

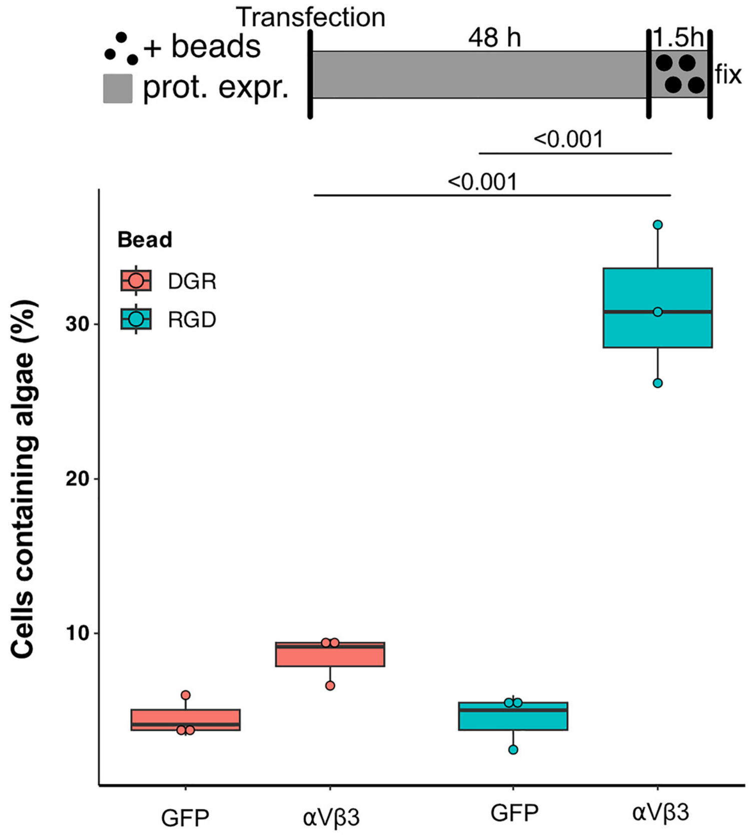

Figure EV4. Upon integrin overexpression, HEK cells preferentially phagocytose RGD-coated beads.

HEK cells transfected with expression plasmids encoding mammalian integrin αV and β3 (each with halves of a split YFP and upon heterodimer formation fluoresce) and exposed to beads coated with either RGD or DGR. Percentage of HEK cells transfected with either αVβ3 integrins or GFP-CaaX as a control that phagocytosed RGD or DGR-coated beads. $n = 3$ biological replicates. Whiskers depict the minima and maxima, the center depicts the median and upper and lower edges of the box depicts the 25th and 75th percentile, respectively. Exact P values are 0.0000577 for the overexpression comparison and 0.0000173 for the RGD GFP and overexpression comparison. Statistical significance was found via ANOVA followed by Tukey.

 © The Author(s)
